# Quantifying microbial growth and carbon use efficiency in dry soil environments via ^18^O water vapor equilibration

**DOI:** 10.1111/gcb.15168

**Published:** 2020-06-24

**Authors:** Alberto Canarini, Wolfgang Wanek, Margarete Watzka, Taru Sandén, Heide Spiegel, Jiří Šantrůček, Jörg Schnecker

**Affiliations:** ^1^ Terrestrial Ecosystem Research Centre for Microbiology and Environmental Systems Science University of Vienna Vienna Austria; ^2^ Department for Soil Health and Plant Nutrition Austrian Agency for Health and Food Safety Vienna Austria; ^3^ Faculty of Science University of South Bohemia Ceske Budejovice Czech Republic

**Keywords:** Birch effect, carbon use efficiency, drought, microbial growth, soil microbial physiology

## Abstract

Soil microbial physiology controls large fluxes of C to the atmosphere, thus, improving our ability to accurately quantify microbial physiology in soil is essential. However, current methods to determine microbial C metabolism require liquid water addition, which makes it practically impossible to measure microbial physiology in dry soil samples without stimulating microbial growth and respiration (namely, the “Birch effect”). We developed a new method based on in vivo ^18^O‐water vapor equilibration to minimize soil rewetting effects. This method allows the isotopic labeling of soil water without direct liquid water addition. This was compared to the main current method (direct ^18^O‐liquid water addition) in moist and air‐dry soils. We determined the time kinetics and calculated the average ^18^O enrichment of soil water over incubation time, which is necessary to calculate microbial growth from ^18^O incorporation in genomic DNA. We tested isotopic equilibration patterns in three natural and six artificially constructed soils covering a wide range of soil texture and soil organic matter content. We then measured microbial growth, respiration and carbon use efficiency (CUE) in three natural soils (either air‐dry or moist). The proposed ^18^O‐vapor equilibration method provided similar results as the current method of liquid ^18^O‐water addition when used for moist soils. However, when applied to air‐dry soils the liquid ^18^O‐water addition method overestimated growth by up to 250%, respiration by up to 500%, and underestimated CUE by up to 40%. We finally describe the new insights into biogeochemical cycling of C that the new method can help uncover, and we consider a range of questions regarding microbial physiology and its response to global change that can now be addressed.

## Graphical abstract

We developed a new method based on in vivo ^18^O‐water vapor equilibration to minimize soil rewetting effects. This method allows the isotopic labeling of soil water without any liquid water or dissolved substrate addition to the sample. When then applied it to dry soils to show that it allows proper estimation of microbial physiology, while the currently widely used method of liquid ^18^O‐water addition overestimated growth by up to 250%, respiration by up to 500%, and underestimated carbon use efficiency by up to 40%. We describe the new insights into biogeochemical cycling of C that the new method can help uncover.

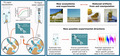

## 1 INTRODUCTION

Soil microorganisms control the rate at which C is released from soils to the atmosphere, but at the same time they regulate soil C sequestration, through microbial growth and death leading to necromass accumulation (Liang, Schimel, & Jastrow, 2017). When retained in the soil, organic C can potentially be stabilized on mineral surfaces (Cotrufo, Wallenstein, Boot, Denef, & Paul, 2013; Kallenbach, Grandy, Frey, & Diefendorf, 2015). Microbial respiration, growth, turnover, and carbon use efficiency (CUE), that is, the proportion of C taken up by microorganisms that is allocated to growth, are key parameters of soil microbial C metabolism. Microbial CUE can be indicative of soil C sequestration (Bradford & Crowther, 2013). Accurate quantification of these parameters and their response to changing environmental conditions is essential for parameterization of models to predict future soil C stocks as well as to develop management practices to promote soil C sequestration (Kallenbach, Wallenstein, Schipanksi, & Grandy, 2019; Li et al., 2019). Under drought, microbial physiology can be altered as soil microorganisms become disconnected from their substrates (de Nijs, Hicks, Leizeaga, Tietema, & Rousk, 2019; Moyano, Manzoni, & Chenu, 2013; Schimel, Balser, & Wallenstein, 2007). Microorganisms must be in contact with soil water to remain active. Because of their semipermeable cell membrane, some microorganisms need to produce osmolytes to reduce their internal water potential and to avoid dehydration and death when soil moisture is low (Borken & Matzner, 2009; Schimel, 2018). Despite these restrictions, soil microorganisms can maintain high levels of activity under drought, as shown by soil respiration and transcriptomics analyses (Roy Chowdhury et al., 2019; Schimel, 2018). Soil microorganisms can maintain activity at much lower water potentials (even lower than −15 MPa) than plants do, as microbial cells interact with smaller soil pores (10–100 µm in size) that might retain hydraulic connectivity under dry conditions, despite negligible diffusivity at the macroscale (Manzoni & Katul, 2014). The different sensitivity of plant CO_2_ assimilation and ecosystem respiration under drought (Schwalm et al., 2010) can cause an ecosystem to turn from a carbon sink into a carbon source (Hoover & Rogers, 2016; Jarvis et al., 2007; Schimel, 2018). While plant responses to drought are relatively well understood, physiological responses of soil microbial communities to drought have remained largely elusive.

**FIGURE 1:**
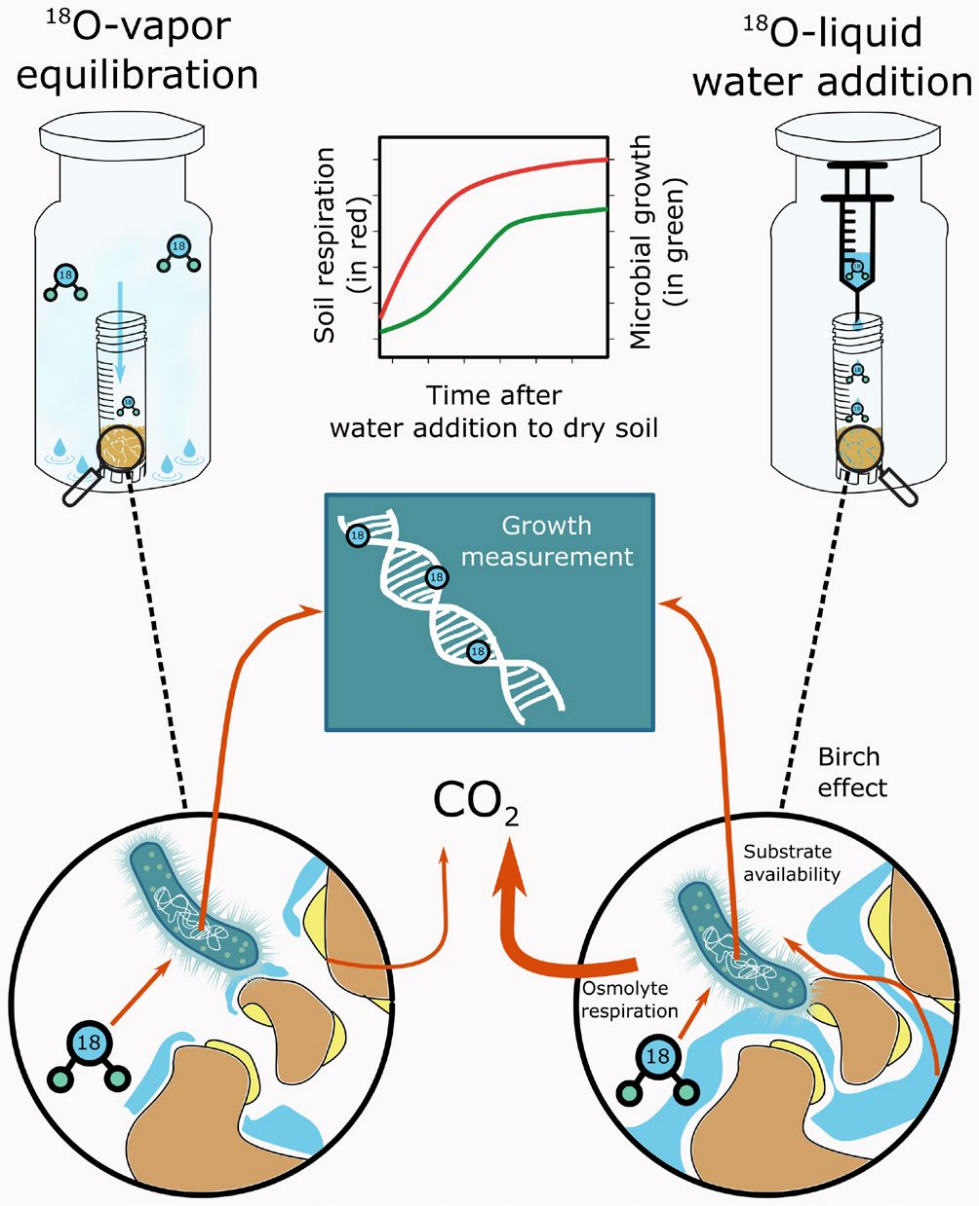
Schematic representation of the different ^18^O methods tested and the start of the Birch effect through the addition of a liquid tracer

The most reliable tools to quantify microbial community‐level physiological processes in soils are based on stable isotope approaches (Dumont & Murrell, 2005). However, classical stable isotope techniques developed for liquid samples or pure cultures fail due to the complexity of the soil matrix. Recently developed methods can quantify essential parameters of microbial physiology, such as microbial growth and CUE (Blazewicz & Schwartz, 2011; Brant, Sulzman, & Myrold, 2006; Spohn, Klaus, Wanek, & Richter, 2016; Zheng et al., 2019). The common ground of these current methods is the addition of ^13^C or ^18^O tracers in liquid form (Geyer, Dijkstra, Sinsabaugh, & Frey, 2019). The addition of ^18^O enriched water, for example, has allowed the substrate independent quantification of microbial growth and CUE (Figure 1), by tracing ^18^O incorporation into genomic DNA (Spohn et al., 2016). However, water added to a dry soil sample causes a burst in microbial activity, as indicated by increases in respiration (up to 500% compared to continuously wet soils; Figure 1). This so called “Birch‐effect” (Birch, 1958) can start minutes after rewetting and last for up to 6 days (Canarini, Kiær, & Dijkstra, 2017; Fraser et al., 2016). The source of this respired C continues to be debated and has been attributed to microbial material (osmolytes or lysed cells), or to mobilization of dissolved organic carbon by aggregate disruption (Canarini et al., 2017; Fraser et al., 2016; Schimel, 2018; Warren, 2014). Similar to respiration, microbial growth is stimulated by re‐wetting after drought, although usually delayed by hours relative to the respiratory response (Blazewicz, Schwartz, & Firestone, 2014; de Nijs et al., 2019; Meisner, Bååth, & Rousk, 2013).

Because of the Birch effect, any method that utilizes water to introduce isotope tracers to study microbial physiology is only able to capture the response of microbial respiration, growth, and CUE to rewetting but not to continuously dry conditions. To our knowledge, only one other study has attempted to develop a method to avoid rewetting effects by applying ^13^C acetic acid vapor (Herron, Stark, Holt, Hooker, & Cardon, 2009). However, addition of ^13^C labeled acetic acid introduces a labile C source that might itself affect microbial growth and respiration (Geyer et al., 2019), and at the same time acetic acid might acidify soils. Here we assessed the validity of a substrate independent method to measure microbial growth in dry soils without changing the soil water content. The method is based on the incorporation of ^18^O into soil water by liquid water‐vapor isotopic equilibration. Because the isotopic composition of two neighboring liquid water pools in a closed environment will approach an average concentration over time (Urey, 1947), ^18^O will equilibrate between the liquid tracer outside the soil and the soil water through evaporation and condensation processes. We assessed the speed of this process and demonstrated the reliability of the method using three different soils by comparison to the currently used methodology (direct ^18^O‐liquid water addition).

## 2 MATERIALS AND METHODS

### 2.1 Sampling sites

Soil samples were collected from three sites: one permanent grassland, one forest, and one agricultural field. The permanent grassland site was sampled in May 2019 and the agricultural site and the forest site were sampled in April 2019. Soil samples were sieved through a 2 mm screen and stored at field moist condition at 4 °C until the start of the experiment in June 2019. For site and soil characteristics, see Table S1.

### 2.2 Experimental setup

Before the experiment, one part of each soil was air‐dried to a water content of around 5% (dry mass basis). The air‐dried soils as well as the moist soils were kept at room temperature to acclimate for 5 days. After that, we carried out three different tests to evaluate:

1. the temporal dynamics of ^18^O equilibration between soil water and vapor;
2. a simplified method to predict temporal dynamics of ^18^O equilibration depending on soil type;
3. the comparability of microbial respiration, growth and CUE obtained by the new ^18^O‐vapor equilibration method and the direct ^18^O‐liquid water addition method.

#### 2.2.1 Temporal dynamics of ^18^O equilibration

To test whether soil water can be sufficiently enriched with ^18^O through water vapor equilibration to allow microbial growth determination in a relatively short time period (24 hr), we set up the following experiment: aliquots (400 mg) of sieved soil were weighed in 1.2 ml plastic vials and inserted in 27 ml glass headspace vials. The headspace vials were sealed air‐tight with rubber septa. This was done for all samples (*n* = 180) including: three soil types, two soil moisture levels (moist and air‐dry), five time points (harvested by shock‐freezing after 2, 4, 8, 16, and 24 hr), two approaches of tracer addition (^18^O‐vapor equilibration vs. the direct ^18^O‐liquid water addition), and three replicates. For the ^18^O‐liquid water addition method, ^18^O labeled water was directly applied to soil, while for the ^18^O‐vapor equilibration method, ^18^O labeled water was externally applied to the bottom of the glass headspace vial with no direct contact to the soil (Figure 1). The amount of water added with the traditional method increased the water content in the soils to 60% of their respective water holding capacity (WHC) and resulted in around 20 atom% ^18^O enrichment in soil water. The same amount of water at the same ^18^O enrichment was also used for the ^18^O‐vapor equilibration method.

#### 2.2.2 Simplified method to indirectly quantify equilibration of ^18^O in soil water

In order to calculate the time kinetics of isotope equilibration in soil water without having to extract the soil water (see Section 2.3), we determined the precision of an indirect measurement. This was achieved with the same experimental conditions as in Section 2.2.1, but only on air‐dry soil samples (as no differences were found in time kinetics between dry and moist conditions) and only for the ^18^O‐vapor equilibration method (*n* = 45). At four time points (2, 4, 8 and 24 hr), the water left at the bottom of the headspace vials was collected and used to analyze its ^18^O enrichment (as in Section 2.3).

#### 2.2.3 Comparison of microbial growth, respiration, and CUE between the two methods

We set up another set of samples to determine microbial growth, respiration and CUE. As in the previous tests, aliquots (400 mg) of sieved soil were weighed in 1.2 ml plastic vials and inserted in 27 ml glass headspace vials. All samples were incubated for 24 hr. Samples included the following: three soils, two soil moisture levels (moist and air‐dry), two approaches of tracer addition (^18^O‐vapor equilibration vs. direct ^18^O‐liquid water addition), and ^18^O labeled versus natural isotope abundance samples in three replicates. To the latter, the same volume of non‐labeled high purity water was added. For the direct ^18^O‐liquid water addition method, the dry samples were also subject to two water additions—either the soil was brought to 60% WHC with ^18^O labeled water (70–160 µl) or we added only 30 μl of ^18^O labeled water (reaching 19%–28% WHC, with a final total enrichment of 20 atom% ^18^O in both cases). The low water addition was done to reduce the effects of rewetting as different moisture conditions between the drying phase and the rewetting phase can influence microbial growth and activity (Canarini et al., 2017; Meisner, Leizeaga, Rousk, & Bååth, 2017). This led to a total of 81 samples. We also incubated three replicates of each soil with no water addition, from which only respiration measurements were taken to assess effects of soil vapor absorption on soil respiration.

After their respective incubation times, one gas sample was collected from each headspace vial to measure CO_2_ accumulation. Then the headspace vials were opened, the plastic vials containing the soil aliquots collected, closed, shock frozen in liquid nitrogen, and stored at −80 °C until further analyses.

### 2.3 Extraction of soil water and determination of its ^18^O enrichment

To determine the ^18^O enrichment in soil water, frozen soil samples from Section 2.2.1 were subjected to cryodistillation, as described in (Plavcova et al., 2018). Briefly, frozen soil samples were transferred to 12 ml glass vials and inserted into a heating block. These were air‐tightly connected to 300 µl plastic vials sitting upside down in a metal block cooled by liquid N. The heating block was heated to 90 °C and the evaporating water was condensed and frozen in the cooled 300 µl plastic vials. To account for potential isotope fractionation during the extraction, water of five different known ^18^O concentrations was treated analogous to the soil samples. Water collected by cryodistillation and from Section 2.2.2 was then analyzed through equilibration of ^18^O in H_2_O with CO_2_ by a Gasbench II headspace sampler connected to a Delta V Advantage isotope ratio mass spectrometer (Thermo Fisher).

### 2.4 Calculation of the soil water ^18^O enrichment in the ^18^O‐vapor equilibration method

As the ^18^O enrichment of the soil water is used to calculate DNA production and ultimately microbial growth (see Equation 2), the average ^18^O enrichment of soil water for the ^18^O‐vapor equilibration method needs to be calculated across experimental time (24 hr), in order to account for the temporal dynamics of isotope equilibration of soil water. To do so, we measured the change in isotopic composition of soil water over time derived from Section 2.2.1. First, we fitted a negative exponential function (Equation 1) as described in Ingraham and Criss (1993) to all the water equilibration treatments as:

(1) $${\;}^{18}O\;{\text{at}\%}_{\text{soil water}}={\;}^{18}O\;{\text{at}\%}_{24}+\left({\;}^{18}O\;{\text{at}\%}_{\text{in}}-{\;}^{18}O\;{\text{at}\%}_{24}\right)*\left(e^{-bt}\right),$$

where ${\;}^{18}O\;{\text{at}\%}_{24}$ and ${\;}^{18}O\;{\text{at}\%}_{\text{in}}$ represent the ^18^O atom % of the soil water after 24 hr incubation and at time point 0, while *b* represents a soil‐specific coefficient that was generated by fitting the *nls()* function in R. Then we calculated the integral of this function by using the function *integrate()* of the R package “pracma” between time 0 and 24 hr. This integral was divided by 24 hr to generate an average isotopic enrichment of soil water for each soil. This number was finally expressed as % of the isotopic enrichment obtained by the direct ^18^O‐liquid water addition method and used in Equation (2) to correct ^18^O at%_soil water_. In order to obtain the average isotopic enrichment of soil water over time for the ^18^O‐vapor equilibration method but avoiding cryodistillation of soil water, we tested samples from experiments described in Section 2.2.2. In this test, ^18^O enrichment was measured in the added isotopically enriched water at the bottom of the headspace vial. The curve obtained here, representing the equilibration of the ^18^O labeled external water via the vapor phase with soil water, was fitted with the same negative exponential model (Equation 1) with the *nls()* function in R, to generate values for the term ${\;}^{18}O\;{\text{at}\%}_{24}$ and *b* (Equation 1) that can be used to predict the isotopic enrichment of the soil water. Predictions were confirmed by plotting values generated from this model on top of the data obtained by measuring ^18^O in water extracted from soils by cryodistillation.

### 2.5 Microbial respiration, growth, and CUE

Microbial respiration, growth, and CUE were determined for samples collected from Section 2.2.3 following the procedures of Spohn et al. (2016) and Zheng et al. (2019) with slight modifications. Microbial respiration was determined by measuring the CO_2_ concentration in the headspace vial right after the application of ^18^O enriched water and 24 hr after the incubation using an infrared gas analyzer (EGM4, PP systems). Microbial growth was determined based on the incorporation of ^18^O from soil water into genomic DNA. DNA was extracted using a DNA extraction kit (FastDNA™ SPIN Kit for Soil, MP Biomedicals). DNA concentration of each extract was determined fluorimetrically following the Picogreen assay using a kit (Quant‐iT™ PicoGreen^®^ dsDNA Reagent, Life Technologies). Subsequently, the ^18^O enrichment and the total O content of the purified DNA fractions were measured using a Thermochemical elemental analyzer (TC/EA Thermo Fisher) coupled via a Conflo III open split system (Thermo Fisher) to an isotope ratio mass spectrometer (Delta V Advantage, Thermo Fisher).

The amount of DNA produced can be calculated using the following equation:

(2) $${\text{DNA}}_{\text{produced}}=O_{\text{DNA}\;\text{extr}}*\frac{{\;}^{18}O\;{\text{at}\%}_{\text{DNA}\;L}-{\;}^{18}O\;{\text{at}\%}_{\text{DNA n.}a.}}{{\;}^{18}O\;{\text{at}\%}_{\text{soil water}}}*\frac{100}{31.21},$$

where O_DNA extr_ is the total amount of oxygen in the DNA extract, ^18^O at%_DNA L_ and ^18^O at%_DNA n.a._ are the ^18^O enrichments in the labeled and unlabeled DNA extracts, respectively, and ^18^O at%_soil water_ is the ^18^O enrichment of the soil water. The fraction at the end of the equation accounts for the average oxygen content in DNA (31.21%). DNA produced can then be used to calculate microbial growth in units of C (Zheng et al., 2019). Soil microbial biomass C, determined by chloroform fumigation extraction (Vance, Brookes, & Jenkinson, 1987), was used to transform DNA amounts into microbial biomass C produced during the incubation. This was done by multiplying DNA_produced_ by the ratio of microbial biomass C:DNA content (f_DNA_) of each soil. The amount of C taken up by the microbial community (C_Uptake_) was estimated as:

(3) $$C_{\text{Uptake}}=C_{\text{Growth}}+C_{\text{Respiration}},$$

where C_Growth_ is the flux of C allocated to biomass production (growth), and C_Respiration_ is the flux of C allocated to the production of CO_2_ (respiration). Microbial CUE was then calculated by the following equation (Manzoni, Taylor, Richter, Porporato, & Ågren, 2012; Sinsabaugh, Manzoni, Moorhead, & Richter, 2013):

(4) $$\text{CUE}=\frac{C_{\text{Growth}}}{C_{\text{Growth}}+C_{\text{Respiration}}}.$$

### 2.6 Statistical analyses

Statistical differences in microbial respiration, growth, and CUE between tracer addition approaches and soil types were assessed by two‐way ANOVA and one‐way ANOVA followed by Tukey HSD post hoc tests. When results were not normally distributed or homoscedastic, data were log or rank transformed using the ARTool package (Wobbrock, Findlater, Gergle, & Higgins, 2011). Statistical analyses were performed in R 3.5.2 (R Core Team, 2017).

## 3 RESULTS AND DISCUSSION

### 3.1 Water equilibration dynamics and adaptation of the ^18^O‐vapor equilibration method

**FIGURE 2:**
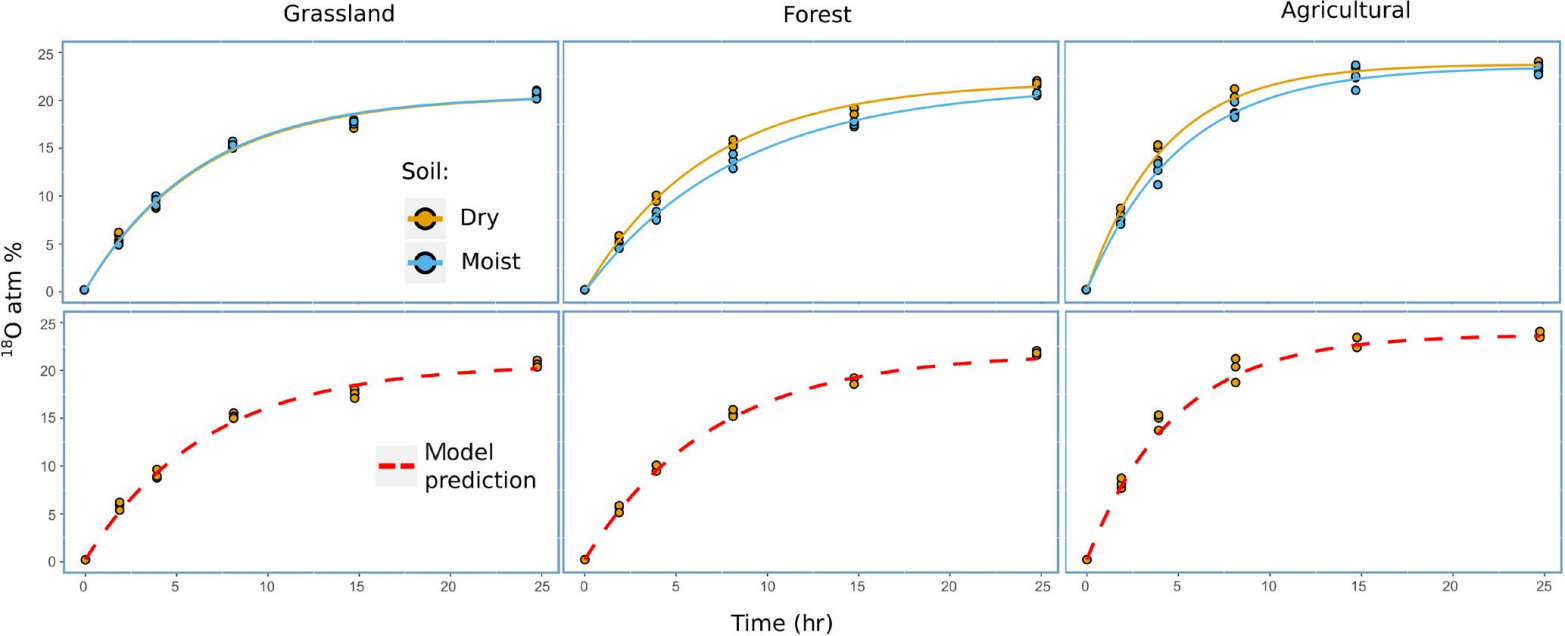
^18^O isotope exchange kinetics of soil water following vapor equilibration (expressed as atom% ^18^O) in the three different soils analyzed (from left to right: grassland, forest, and agricultural soil) with incubation time (*x*‐axis). In the upper panels points represent data obtained from cryodistillation extraction of soil water for two soil moisture conditions (air‐dry and moist soils) and lines represent the best model fit from Equation (1). The bottom panels represent the model prediction generated from measurements of ^18^O kinetics in the labelled external water pool during isotope exchange with soil water via vapor exchange as described in Sections 2.2.2, 2.3 and 2.2.3, 2.4 of Materials and Methods for dry soils only.

**FIGURE 3:**
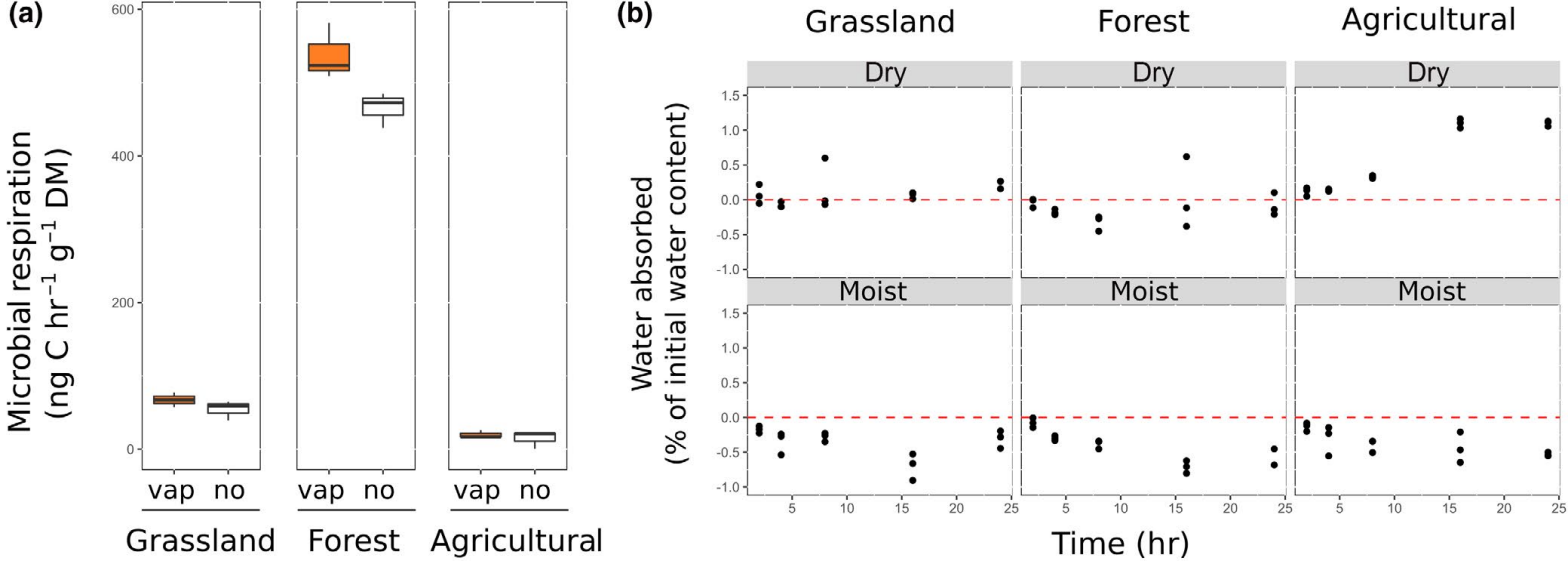
(a) Respiration rates of grassland, forest, and agricultural soils treated with the ^18^O‐vapor equilibration method (orange boxes) compared to untreated soils with no direct or indirect water application (white boxes). No significant differences were found following a two‐way ANOVA test (*F* = 1.461; *p* = .25). Values were log‐transformed to meet the assumption of homogeneity of variances. (b) Soil vapor absorption (net weight gain) or soil evaporation (net weight loss) in the three tested soils (grassland, forest, and agricultural soil) at the different soil moisture levels (air‐dry, moist) used during the experiment (calculations are described in the Supplementary Methods section). Negative values indicate no net vapor absorption but rather soil evaporative water loss from moist soils while dry soils gained weight by vapor absorption. This net gain ranged between 0% in dry forest soils, 0.1% in dry grassland soils, and 1.1% in dry agricultural soils (all percentages given relative to initial soil water content). Net gains through soil vapor absorption were therefore negligible.

In a closed system of two liquid water sources, the oxygen isotopes will redistribute via the vapor phase to a common concentration. Here we tested whether the ^18^O‐vapor equilibration method allows isotopic enrichment of soil water to a similar extent as direct ^18^O‐liquid water addition (20 at%^18^O) within a relatively short time period (24 hr). Figure 2 (top panels) shows the ^18^O isotope exchange kinetics of soil water following vapor equilibration in the three different soils, and the negative exponential model (Equation 1) fitted to the data. The results show that ^18^O enrichment of soil water reached values equivalent to those of direct ^18^O liquid water addition (~20 at%^18^O) within 24 hr. Very similar equilibration rates were found for air‐dry and moist soils, while the ^18^O equilibration rates differed between soil types. In an ideal situation, the isotopic exchange between two water pools, that is, an internal soil water pool and an external ^18^O water pool, through the vapor phase is controlled by the relative pool sizes of the water pools, the isotopic composition of the water pools involved, and the relative surface areas of these water pools (Ingraham & Criss, 1993). However, soil properties may also affect isotopic exchange rates. For instance, larger aggregate sizes and/or a higher soil porosity may lead to higher water vapor diffusion rates into soils (Jabro, 2009). We therefore investigated different soil properties (including soil texture, soil organic matter content, soil vapor sorption) that might explain the observed differences in ^18^O exchange rates (see Supplementary Methods and Figures S1 and S2 and Figure 3b) but found no relation with any of the soil properties tested.

We then assessed the use of an indirect measurement of ^18^O enrichment kinetics in soil water, in order to calculate isotope exchange rates without the necessity to extract soil water by cryodistillation. We replicated the same experimental set up as used to determine isotopic exchange in cryodistilled soil water for dry soil samples, but collected and isotopically characterized the external isotopically labeled water pool from the bottom of the head space vial at different time points. Thus, we obtained isotopic equilibration rates of the external ^18^O water source (Figure S3). By fitting Equation (1), we then calculated the coefficient *b* of Equation (1). By knowing the initial at%^18^O values of soil water and final values after 24 hr, we generated a model that was able to fit the data obtained by direct soil water extraction (Figure 2, bottom panels). The model was tested against the best fit model, and led to very similar model fittings (see Figures S3 and S4 and Table S2) also when used for moist soil conditions (Figure S4). This allowed us to adopt a rapid and simple method to indirectly calculate the time‐averaged ^18^O enrichment of soil water that soil microorganisms are exposed to during the experiment. These values need to be measured for each soil type before an experiment, and also if headspace vials with different volume are used, as this might affect the ^18^O equilibration time kinetics.

### 3.2 Methodological differences in microbial growth, respiration, and CUE estimates

**FIGURE 4:**
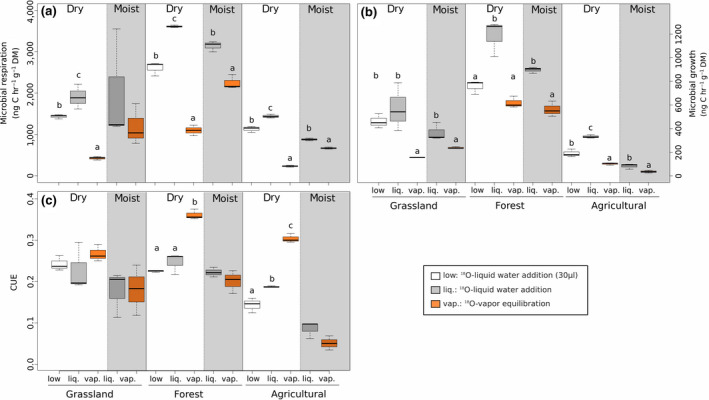
Soil microbial respiration (a), growth (b), and carbon use efficiency (CUE; c) of a grassland, a forest, and an agricultural site measured in air‐dry and moist soils using direct ^18^O‐liquid water addition at high water addition rate (increasing soil water content to 60% water holding capacity; grey boxes) or with minimal liquid ^18^O‐water addition (white boxes; dry soils only), and using ^18^O‐vapor equilibration (orange boxes). Letters indicate statistically significant differences. See Table S3 for full statistical results.

By accounting for the time kinetics of ^18^O equilibration between external labeled water and soil water (as explained in Section 3.1) we calculated microbial growth in the ^18^O‐vapor equilibration method. Microbial growth, respiration, and CUE could thus be compared between ^18^O‐vapor equilibration and direct ^18^O‐liquid water addition. As expected, dry soils to which water was added directly showed a strong increase in microbial respiration. Dry soils subjected to the ^18^O‐vapor equilibration method respired three to six times less than rewetted soils (Figure 4a), which had values 346%, 229%, and 516% higher in the grassland, forest, and agricultural site, respectively. We also measured respiration without any addition of water and found no significant difference in respiration rates between untreated soils and soils undergoing vapor equilibration (Figure 3a). However, recent studies have highlighted that in some ecosystems (e.g., arid ecosystems), non‐rainfall moisture (high humidity, dew, and fog) can significantly increase microbial activity (Evans, Todd‐Brown, Jacobson, & Jacobson, 2019). The increase in respiration rates following water addition to dry soils is a common phenomenon (Birch, 1958). While the exact nature of the C released through respiration upon rewetting is still subject to debate, it is known to derive from a combination of abiotic processes, internal use of metabolites, and increased reconnection between available substrates and decomposers (Schimel, 2018; Schimel et al., 2007). Interestingly, also moist soil obtained from the forest and agricultural sites respired 30%–40% more when water was directly added compared to the ^18^O‐vapor equilibration method, but not in the grassland soil. Because different soils have different soil moisture ranges at which microorganisms reach optimum activity (Moyano et al., 2012), this result might indicate that adding water causes increases in microbial activity only when the initial soil water content is lower than optimal. Similar to respiration, rewetting of dry soils stimulated microbial growth (Figure 4b). Microbial growth was always higher when water was added directly compared to the ^18^O‐vapor equilibration method. The differences were significant in all soils, all water regimes, and for all water amounts added, with the exception of dry forest soils. Here only the larger water addition (increasing soil water content to 60% WHC) showed significantly higher growth rates than the ^18^O‐vapor equilibration method (for full statistical results see Table S3). We could thus show that adding water to dry soils can cause misleading results if used to estimate microbial growth, as microbial growth is known to increase following rewetting of dry soils for both, bacteria and fungi (Hicks, Ang, Leizeaga, & Rousk, 2019). This increase in growth rates following rewetting had values 279%, 92%, and 226% higher in the grassland, forest, and agricultural soil, respectively.

We found no difference in microbial CUE between both methods in moist soils of all sites (Figure 4c). This shows that although growth and respiration may be stimulated by liquid water addition in fresh soils, their increase was proportional and therefore did not affect CUE estimates in moist soils. On the other hand, when liquid water was directly added to dry soils microbial CUE was significantly underestimated compared to the ^18^O‐vapor equilibration method in forest and agricultural soils (32% and 38% lower, respectively). This indicates that when water is added directly to dry soils, the response of microbial growth and respiration was not proportional. In dry soils the stronger stimulation of respiration than growth by water addition underestimated microbial CUE. Moreover, we found that microbial CUE was significantly higher in dry compared to moist soils (measured by ^18^O‐vapor equilibration method), indicating a greater sensitivity of respiration than growth to soil drying.

## 4 CONCLUSIONS

Microbial physiology controls large fluxes of C from soil to the atmosphere but also the proportion of C remaining in the soil that can potentially be stabilized. Microbial growth, respiration, and CUE thus require precise quantification to improve predictions of soil C cycling. A caveat of current approaches to measure microbial physiology is that a tracer is introduced with an aqueous solution, inevitably causing rewetting of dry soils. Here we present a new approach (^18^O‐vapor equilibration) that resolves this issue and expands the possibilities of future studies to accurately quantify microbial growth and CUE in dry soils. The proposed method uses isotopic equilibration between an external ^18^O labeled water pool and soil water via the vapor phase and provides similar microbial CUE results as the direct ^18^O liquid water addition method when used at near‐optimal soil water content. However, when applied to dry soil the liquid water addition overestimated microbial growth by up to 250%, respiration by up to 500%, and underestimated CUE by up to 40%. The ^18^O‐vapor equilibration method thus greatly reduces rewetting biases. We further describe new insights into the biogeochemical C cycle that the new method can help uncover (Box 1) and consider a wide range of questions regarding microbial physiology and its response to global change that can now be proposed and addressed.

BOX 1 **Potential applications of the ^18^O‐vapor equilibration method to investigate microbial community growth and carbon use efficiency (CUE) in a range of different ecosystems and experimental approaches.**

The benefit of a substrate‐independent technique (^18^O‐enrichment of soil water) combined with vapor equilibration is the reduction of rewetting artifacts caused by introducing an isotopic tracer by direct liquid water addition. This enables to expand measurements of microbial community level growth and CUE to a range of new ecosystems (i.e., drylands) and to experimental manipulations (i.e., drought experiments). Since the ^18^O‐vapor equilibration method does not require the homogeneous distribution of the liquid tracer into the soil medium (vapor equally penetrates the soil), the technique can also be applied to intact soil cores, further reducing artifacts introduced by soil handling (e.g., intact biocrust samples where root removal is not needed). This combined with the qSIP method (H_2_^18^O quantitative stable isotope probing; Hungate et al., 2015) could enable to generate for the first time taxon‐specific growth rates in undisturbed dry soil environments.

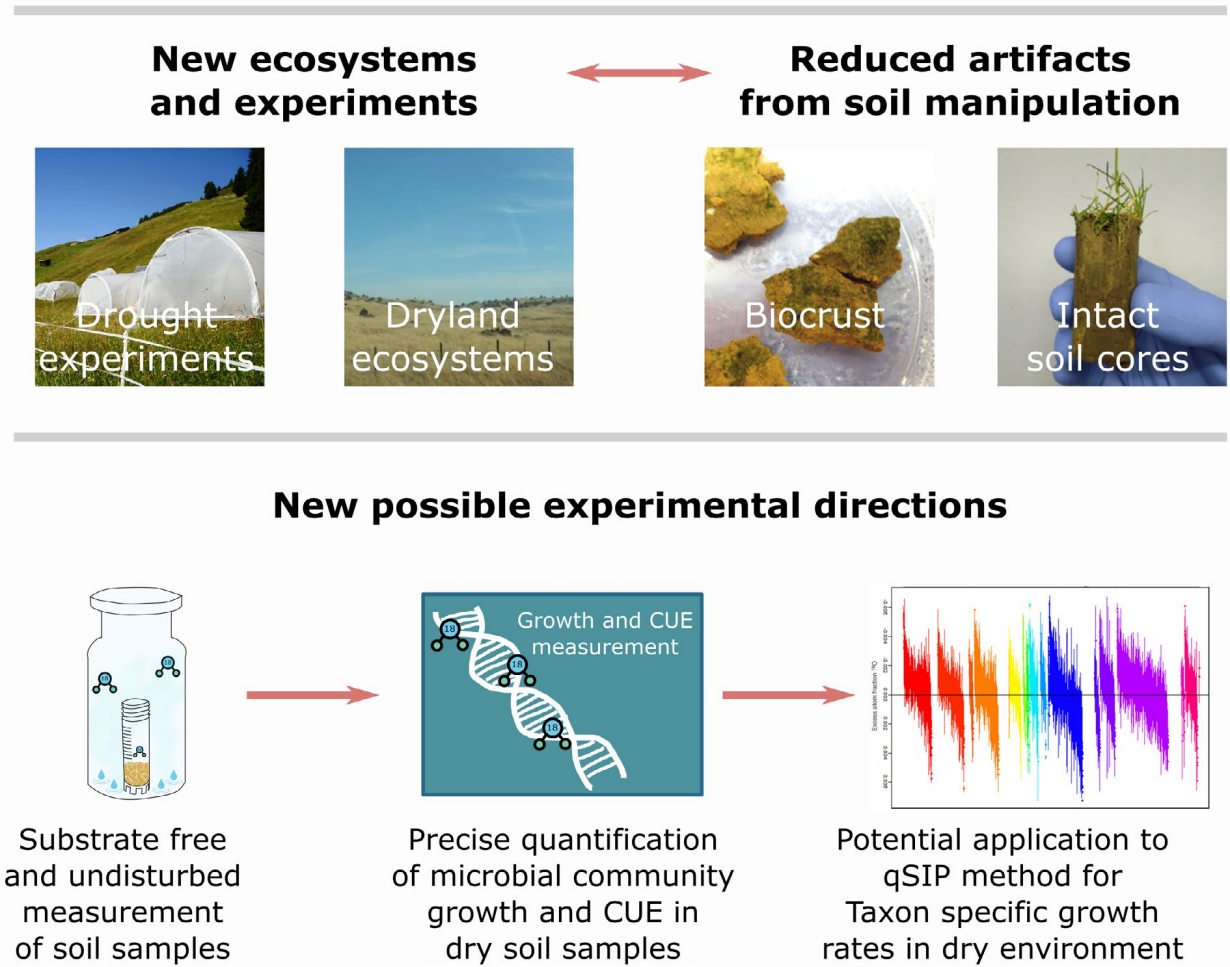

## Supporting information

Supplementary Material

## Data Availability

The data that support the findings of this study are available from the corresponding author upon reasonable request.
