# Supplementary material for: Quantifying microbial growth and carbon use efficiency in dry soil environments via 18O water vapor equilibration
Source: Glob Chang Biol. 2020 Jun 24;26(9):5333–41. doi: 10.1111/gcb.15168 (PMC7497233; doi:10.1111/gcb.15168)
Supplement: Supplementary file 1 — Supplementary Material [file GCB-26-5333-s001.pdf]

Supplementary methods:

In order to understand soil properties that could explain soil-specific differences in ^{18}O equilibration rates between the vapor phase and the soil water, we tested the effect of three edaphic properties:

1) Soil texture
2) Soil organic matter content
3) Soil vapor absorption

Soil texture:

We created three artificial soils and tested ^{18}O water equilibration dynamics to compare with the soils under study (grassland, forest and agricultural soil). The three artificial soils contained a mix of pure silt and clay (montmorillonite) mixed with pure sand in three different proportions: 30% sand (C); 50% sand (CS) and 70% sand (S). The clay and silt and the sand fractions contained no organic carbon. Soils were maintained at 20% water content (measured gravimetrically). Aliquots (400 mg) of soil were weighed in 1.2 mL plastic vials and inserted in 27 mL glass headspace vials. The headspace vials were sealed air-tight with rubber septa. Analyses were carried out in triplicates. The ^{18}O labelled water was applied at the bottom of the glass headspace vial, as explained in the materials and methods and the amount of water added followed the same calculations. Soil water was extracted via cryo-distillation at different time points (2, 4, 8, 16, and 24 hours) and the extracted water was analyzed for ^{18}O enrichment as described in the Materials and Methods section. Results are shown in Supplementary Figure S1.

Soil organic matter content:

We created three additional artificial soils and tested ^{18}O vapor equilibration dynamics to compare with the soils under study (grassland, forest and agricultural soil). The three artificial soils contained a mix of the forest soil with artificial soil (50% sand, 50% silt and clay) which were mixed in different proportion: 100% forest soil (H); 50% forest soil (M); 25% forest soil (L). The experiment was replicated as described for the soil texture experiment. Results are shown in Supplementary Figure S2.

Soil vapor absorption:

The three soils under study were subjected to the same settings as described in section 2.2.1 of the Materials and Methods section, but only for the ^{18}O vapor equilibration. Before incubation vials and soil weights were recorded to the nearest µg. After the incubation the vials were removed and weighed in order to calculate the amount of water absorbed or lost by evaporation during the incubation. Results are shown in the main text Figure 3.

Statistical analysis

To assess effects of texture and organic matter on ^{18}O water equilibration we used a linear mixed effect model with the function *lme* from the package nlme, to test the interactive effects of treatment and time. Time was also included as a random factor nested within each replicate to account for the non-independence of time replicates. To assess effects of water vapor on soil respiration rates compared to no water addition we used a two-way ANOVA to assess effects of site and treatment. Results from F-tests were generated with the function *anova* and are reported in each figure legend.

Supplementary Figures:

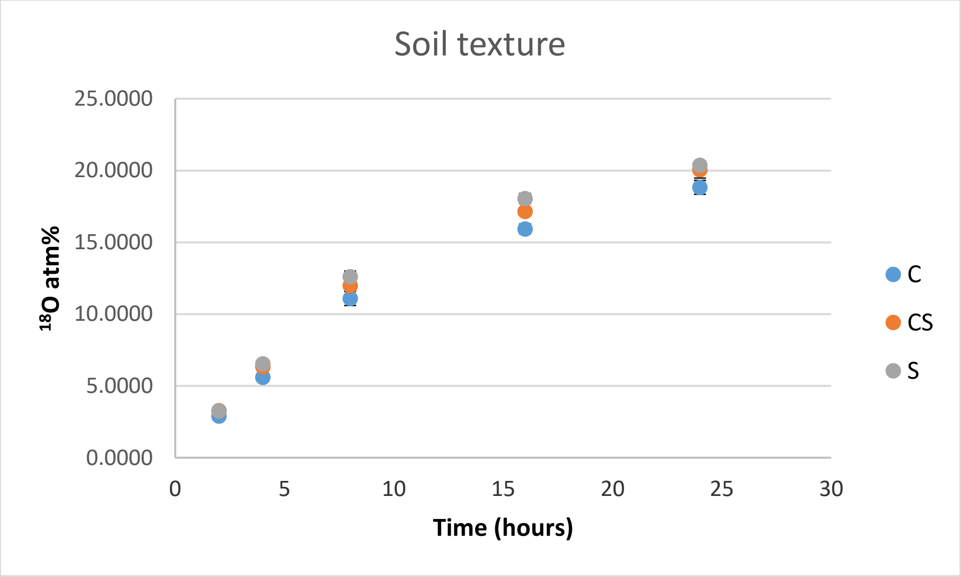

Figure S1. Testing soil texture effects on ^{18}O equilibration dynamics between external ^{18}O labelled water and soil water through the vapor phase in the three artificial soils: C (30% sand), CS (50% sand) and S (70% sand). Equilibration dynamics did not differ significantly between the three artificial soils (F= 0.88; p= 0.46).

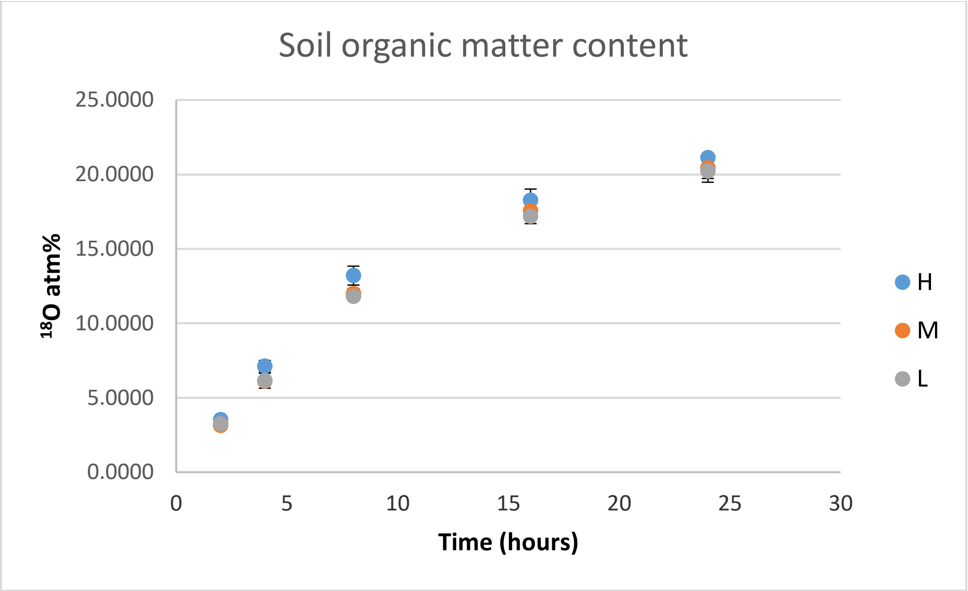

Figure S2. Testing soil organic matter content effects on ^{18}O equilibration dynamics between external ^{18}O labelled water and soil water through the vapor phase in three artificial soils. The soils represented mixtures of native forest soil with increasing proportions of artificial soil (50% sand, 50% silt and clay). H (100% forest soil), M (50% forest soil) and L (25% forest soil). Equilibration dynamics did not differ significantly between the three soils (F= 0.44; p= 0.66).

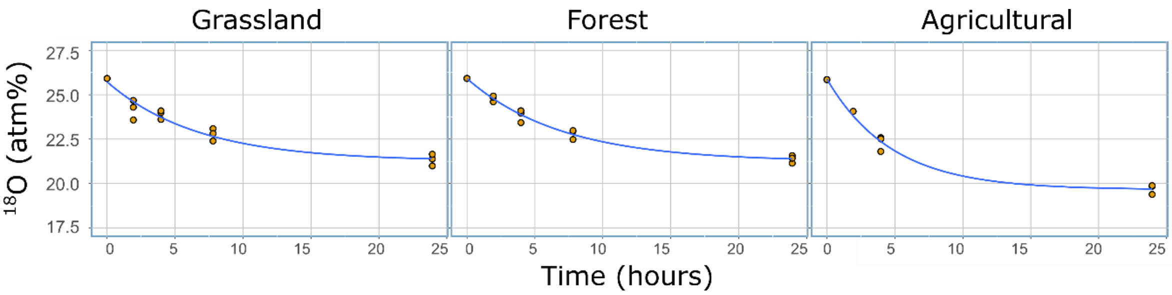

Figure S3. Isotopic equilibration rates of the external ^{18}O labelled source water with soil water of the three soil types (grassland, forest and agricultural) under air-dry conditions.

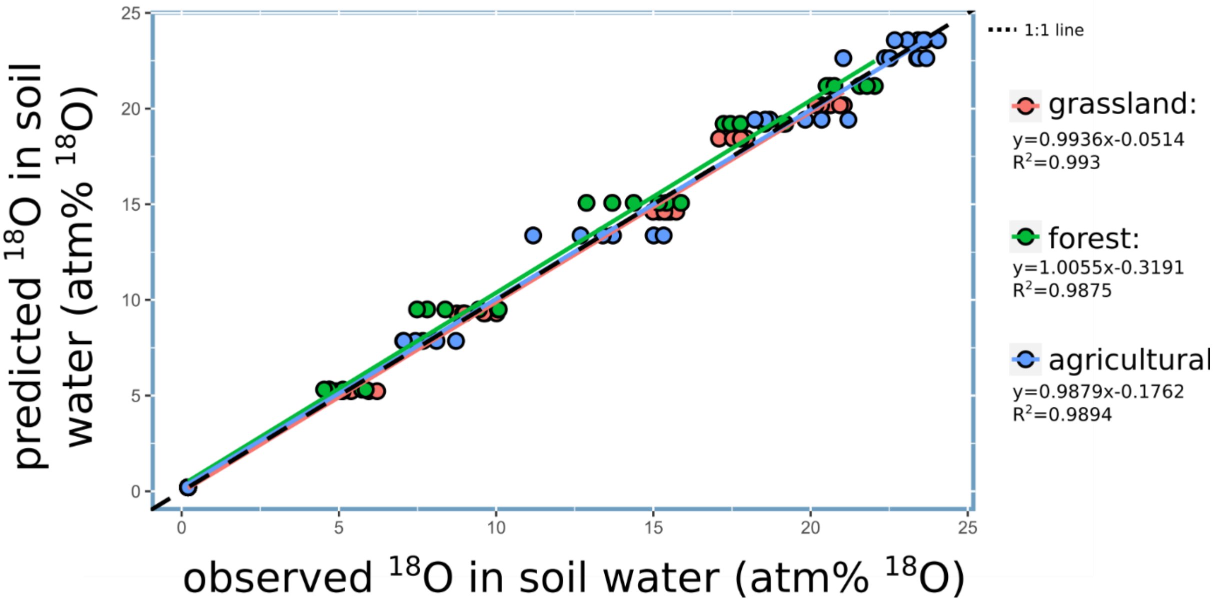

Figure S4. Observed vs predicted ^{18}O enrichment values in soil water for the three soil types (grassland, forest, agricultural) including air-dry and moist soils. Observed values are from soil water extracted by cryo-distillation, predicted values derive from the model fitted using ^{18}O enrichment measurements in the labelled water added externally. The dashed line represents the 1:1 line. Regression equations and R^2 values are reported.

Supplementary Tables:

Table S1: Soil and site characteristics

**Variable**		**Site**		
		Grassland	Forest	Agricultural
Location		47°29'N, 14°6'E	47°42'N, 16°17'E	48°12´N 15°15´E
pH		5.5	4.9	6.1
SOC %		2.4	4.3	0.9
Sand %		40	55	10
Silt %		51	38	73
Clay %		9	7	17
Water content before incubation (% dry soil)	moist	26.9	20.8	15.2
	dry	5	7	3.2
Mean annual temperature (°C)		8.4	6.5	8.5
Mean annual precipitation (mm)		1015	800	840
Soil type		Cambisol	pseudo-gleyic Cambisol	Gleyic Luvisol
Texture*		Silt loam	Sandy loam	Silt loam

*according to the USDA soil taxonomy

Table S2: Model estimates comparing the best model generated from the data and the model predictions of ^{18}O equilibration rates.

Soil:	Grassland		Forest		Agricultural	
	**Model**					
**Comparison estimates**	Estimated	Predicted	Estimated	Predicted	Estimated	Predicted
AIC	41.65	42.54	24.16	30.35	42.27	97.27
BIC	45.21	46.10	27.72	33.91	45.83	100.82
Log-likelihood	-16.83	-17.27	-8.08	-11.17	-17.13	-44.63
**Coefficients**						
$^{18}O\ at\%_{24}$	20.66	20.68	22.08	21.80	23.78	23.70
$^{18}O\ at\%_{in}$	0.42	0.20	0.32	0.20	0.10	0.20
b	0.15	0.15	0.14	0.14	0.23	0.21

Table S3: Results from ANOVA testing for the effect of water application approach and post-hoc tests corresponding to Figure 3 (main text). Comparisons between application type (“d” is direct water application to reach 60% WHC, “l” is reduced direct water application, and “i” is (indirect) vapor equilibration) for the individual combinations of soil and water content.

		CUE			Respiration			Growth		
		F	p-value	post-hoc	F	p-value	post-hoc	F	p-value	post-hoc
Grassland	moist	0.003	0.957		0.943	0.387		8.548	0.043	d>i
Forest	moist	1.544	0.282		50.99	0.002	d>i	69.13	0.001	d>i
Agricultural field	moist	5.122	0.086		86.09	0.001	d>i	10.02	0.034	d>i
Grassland	dry	1.047	0.407		54.15	>0.001	d>l>i	9.292	0.015	d>i
Forest	dry	59.66	>0.001	i>d,l	323.9	>0.001	d>l>i	27.27	0.001	d>i,l
Agricultural field	dry	143	>0.001	i>d>l	436	>0.001	d>l>i	88.45	>0.001	d>l>i
